# Pain Severity and Smoking Abstinence Expectancies among Latinx Individuals Who Smoke Cigarettes: The Moderating Role of Perceived Discrimination

**DOI:** 10.3390/ijerph20021079

**Published:** 2023-01-07

**Authors:** Brooke Y. Redmond, Aniqua Salwa, Tanya Smit, Joseph W. Ditre, Lorra Garey, Michael J. Zvolensky

**Affiliations:** 1 Department of Psychology, University of Houston, Houston, TX 77004, USA; 2 Department of Psychology, Syracuse University, Syracuse, NY 13244, USA; 3 Department of Behavioral Science, The University of Texas MD Anderson Cancer Center, Houston, TX 77030, USA; 4 Health Institute, University of Houston, Houston, TX 77004, USA

**Keywords:** pain severity, smoking abstinence expectancies, Latinx/Hispanic, tobacco, perceived discrimination

## Abstract

Latinx individuals experience significant health disparities related to smoking cessation in the United States (US). Although past works have consistently implicated pain in the maintenance of smoking behavior, limited research has examined the role of social determinants (e.g., perceived discrimination) in pain–smoking relations. The current study sought to examine the moderating role of perceived discrimination in the relation between pain severity and smoking abstinence expectancies (i.e., a cognitive factor related to poor smoking outcomes) among 226 Latinx individuals who currently smoke cigarettes (*M_age_* = 34.95 years; *SD* = 8.62; 38.5% female). The results indicated a statistically significant interaction between pain severity and perceived discrimination with regard to smoking abstinence expectancies (i.e., negative mood, somatic symptoms, harmful consequences, and positive consequences). Post-hoc analyses revealed the association of pain severity and negative mood, harmful consequences, and positive consequences smoking abstinence expectancies evident for individuals with higher perceived discrimination. Moreover, the association between pain severity and somatic symptoms smoking abstinence expectancies was stronger for individuals with higher perceived discrimination. Overall, these results suggest that clinical and community-based public health strategies may benefit from addressing the role of perceived discrimination among Latinx individuals who smoke cigarettes in the context of pain.

## 1. Introduction

Latinx individuals experience significant health disparities related to smoking cessation services and outcomes in the United States (US) [1,2,3]. For example, compared to other racial/ethnic groups, Latinx individuals are less likely to receive advice from health-care professionals on quitting smoking [4]. Similarly, compared to non-Latinx White individuals who smoke cigarettes, racial/ethnic minorities (i.e., Latinx and African American) who smoke cigarettes are less likely to use efficacious smoking cessation treatments, such as one-on-one counseling (e.g., Quitline and stop smoking class) and pharmacotherapy (e.g., varenicline) [3,5]. The current smoking cessation approaches are not meeting the needs of Latinx individuals, particularly those with comorbid symptoms/conditions

Co-occurring pain is one behavioral health factor that is consistently related to cigarette smoking [6,7,8]. Among individuals in the general population, pain has been related to increased cigarette consumption and greater tobacco dependence [6,9]. Extant research further indicates that Latinx individuals tend to score higher on measures of pain severity, pain-related anxiety, and pain catastrophizing than non-Latinx White individuals [10,11]. Latinx individuals have also been shown to experience more severe pain before seeking treatment than non-Latinx White individuals [12]. Importantly, more severe and distressing pain has been linked with the employment of cigarette smoking as a pain-coping strategy [13,14]. In line with this perspective, Latinx individuals with co-occurring pain who smoke tend to report elevations on measures of tobacco dependence, perceived barriers to quitting, and difficulty experienced when attempting to quit [15]. Thus, there is a clear need to examine the role of pain severity in relation to other clinically important processes linked to the maintenance of smoking among Latinx individuals.

Smoking abstinence expectancies (i.e., the appraisal of anticipated outcomes from refraining from cigarette smoking) have long been linked to cessation-relevant outcomes [16,17], including success in quitting [18], cigarette dependence [19], motivation to quit [20], and nicotine withdrawal [21]. Individual smoking abstinence expectancies relate to negative mood (e.g., “I would feel tense”), somatic symptoms (“My chest would feel tight”), harmful consequences (e.g., “I would feel like I’m dying”), and positive consequences (e.g., “I would feel calm”) [22]. The initial works, conducted among largely non-Latinx White samples, showed that individuals with co-occurring pain who smoke cigarettes hold expectancies for more severe nicotine withdrawal than individuals without co-occurring pain who smoke cigarettes [23]. Similar pain–abstinence expectancy relations have also been observed in the context of e-cigarette use [24]. Importantly, this work has not yet been extended to Latinx individuals who smoke cigarettes.

When considering the association between pain severity and smoking abstinence expectancies among Latinx individuals, it is also important to consider the role of social determinants of health that may further intensify these relations. Perceived discrimination (i.e., reported experiences of unfair treatment based on race or ethnicity) [25] is a clinically important construct in terms of the impact on health-related behaviors [26]. Perceived discrimination can involve reported experiences of social distancing and stigmatization, among other experiences (e.g., harassment, violent acts, etc.) [25,27]. Among Latinx individuals, perceived discrimination is common (e.g., 80% past month prevalence) [28], with some works suggesting rates of occurrence as high as 94.2% [29]. Importantly, prior research has linked perceived discrimination with poorer smoking outcomes [30,31,32,33]. For example, Kendzor et al. [32] found experiencing a greater number of major discrimination events was associated with a reduced likelihood of achieving 7-day point prevalence smoking abstinence and continuous smoking abstinence. However, no work to date has extended this model to examine whether perceived discrimination may interact with pain severity to influence smoking abstinence expectancies among Latinx individuals who smoke cigarettes. 

Theoretically, perceived discrimination may contribute to deleterious pain trajectories and problematic smoking abstinence expectancies (e.g., negative mood, somatic symptoms, and harmful and positive consequences). For example, Carlisle [34] found that perceived discrimination was associated with a greater likelihood of reporting a chronic pain condition among Latinx individuals, which appears to be consistent with evidence that the accumulation of discriminatory experiences among Latinx individuals is associated with a marker of heightened pain facilitation (i.e., temporal summation of pain) [29]. Other works have shown that psychological distress due to perceived discrimination is associated with a greater probability of experiencing chronic pain [35]. Given that Latinx individuals have endorsed a perception that healthcare providers do not care about or believe their reports of pain [36], these individuals may engage in cigarette smoking in an effort to cope with pain [13]. Over time, these individuals may begin to form distinct beliefs about the anticipated effects of smoking abstinence.

The current study sought to test the interactive association between perceived discrimination and pain severity in relation to smoking abstinence expectancies among Latinx individuals who smoke cigarettes. It was hypothesized that pain severity would be positively associated with negative mood, somatic symptoms, and harmful consequences abstinence expectancies and that these associations would be stronger at higher levels of perceived discrimination. It was also hypothesized that pain severity would be negatively associated with positive abstinence expectancies and that the association would be stronger at higher levels of perceived discrimination. Finally, we anticipated that these relations would remain evident after controlling for relevant covariates, including age [37,38], sex [39,40], nativity (i.e., place of birth) [41], income [42,43], education [43], and number of cigarettes smoked per day [44,45]. 

## 2. Methods

### 2.1. Participants

Participants in the current study included a nationally representative sample of 226 adult Latinx individuals who engage in daily cigarette use (*M_age_* = 34.95 years; *SD* = 8.62; 38.5% female). The majority of participants (86.3%) were born in the US. The racial breakdown of participants included: 69% White, 10.6% Other, 9.3% Black or African American, 5.3% Alaska Native or American Indian, 2.6% More than one race, 1.3% Asian, and 1.3% Native Hawaiian or Other Pacific Islander (0.4% did not respond). Participants reported smoking an average of 12.15 (*SD* = 10.37) cigarettes per day and reported daily cigarette smoking for an average of 14.57 years (*SD* = 9.57). Participants average age of onset as a daily cigarette smoker was 20.37 (*SD* = 4.41) years old, and they reported, on average, 5.65 (*SD* = 7.93) serious attempts to quit smoking. Pain severity measured via the Brief Pain Inventory-Short Form pain severity subscale (BPI-SF) [46] was characterized utilizing established cut-offs (i.e., mild = 1–4, moderate = 5–6, and severe = 7–10) [47] and indicated 65 participants (28.8%) met the criteria for mild pain, 95 (42.0%) met the criteria for moderate pain, and 66 (29.2%) met the criteria for severe pain. 

### 2.2. Measures

*Demographics Questionnaire.* A demographics questionnaire was used to assess participant age, sex assigned at birth, nativity, income, and education.

Smoking History Questionnaire (SHQ) [48]. The SHQ is a measure of smoking behavior (e.g., cigarettes smoked per day) and the severity of problems experienced during prior quit attempts. 

Brief Pain Inventory-Short Form (BPI-SF) [46]. The BPI-SF is a 9-item form that includes a measure of pain severity. Pain severity was measured by four items (e.g., “Please rate your pain by marking the number that best describes your pain in the last 24 h”) on a scale from 0 (no pain) to 10 (pain as bad as you can imagine). The pain severity subscale demonstrated good internal consistency in the current study (α = 0.89), as in past works [49]. 

Brief Perceived Ethnic Discrimination Questionnaire-Community Version (PEDQ-CV) [25]. The Brief PEDQ-CV is a 17-item multidimensional self-reporting measure of perceived racism and ethnic discrimination adapted from the original 70-item PEDQ [27]. The Brief PEDQ-CV measures five factors: (1) Lifetime exposure, (2) Exclusion/Rejection, (3) Stigmatization/devaluation, (4) Discrimination at work/school, and (5) Threat/aggression. Participants are asked to rate each item from 1 (Never happened) to 5 (Happened very often). Items are averaged to create a total score. The Brief PEDQ-CV has demonstrated sound psychometric properties in past works [25]. In the current study, the Brief PEDQ-CV demonstrated excellent internal consistency (α = 0.96).

Smoking Abstinence Expectancies Questionnaire (SAEQ) [22]. The SAEQ is a 28-item measure of psychological and physiological consequences expected from smoking abstinence. Participants are asked to rate items on a 7-point Likert scale ranging from 0 (Very unlikely) to 6 (Very likely). The SAEQ yields four subscales. Three of the subscales represent negative abstinence expectancies, including negative mood (e.g., “I would feel tense”), somatic symptoms (e.g., “My chest would feel tight”), and harmful consequences (e.g., “I would feel like I’m losing control”). A fourth subscale reflects positive expectancies (positive consequences, e.g., “I would feel happy”). The SAEQ has demonstrated good psychometric properties in past works, including internal consistency, test–retest reliability, and convergent and discriminant validity [18,20,22]. The four subscales were used as criterion variables in the current study, and all demonstrated excellent internal consistency (α’s range = 0.90–0.91). 

### 2.3. Procedure

Participants were recruited nationally across the US using Qualtrics Panels. Qualtrics Panels is an online survey management system that has been used successfully in prior research to target specific populations and gather valid and reliable data [50,51]. Participants with a Qualtrics Panels account who identified as Latinx and endorsed current cigarette smoking were sent a study advertisement. Interested respondents were then screened for eligibility and directed to complete a survey. Informed consent was obtained from all participants. Eligibility criteria for the current study included being at least 18 years of age, identifying as Latinx, and self-reporting current daily cigarette smoking (> 5 cigarettes per day). Compensation for participation was equivalent to USD $10.75. Participants could opt to receive their compensation via cash-based incentives (i.e., gift cards), reward miles, or reward points. For quality assurance, a variety of methods were utilized, including a speeding check (i.e., one-half the median survey completion time), as well as additional safeguards to prevent multiple attempts to complete the survey by the same respondent (i.e., recording IP addresses). The study was approved by the Institutional Review Board of the university where the study took place.

### 2.4. Analytic Strategy

Analyses were conducted using SPSS version 28. First, descriptive statistics and bivariate correlations among the study variables were examined. A point–biserial correlation was examined between sex and all other study variables. The effect sizes for correlations among the study variables were interpreted as weak (.10), moderate (.30), or strong [50,52]. Next, to test the main and interactive effects of pain severity and perceived discrimination on smoking abstinence expectancies, four hierarchical linear regression analyses were conducted for each of the criterion variables: (1) negative mood abstinence expectancies, (2) somatic symptom abstinence expectancies, (3) harmful consequences abstinence expectancies, and (4) positive consequences abstinence expectancies. Step 1 for all analysis included covariates of age; sex (Coded: 0 = Male, 1 = Female); nativity (Coded: 0 = US Born, 1 = Born Outside of US); income (Coded 1 = USD $0–$4999, 2 = USD $5000–$9999, 3 = USD $10,000–$14,999, 4 = USD $15,000–$24,999, 5 = USD $25,000–$34,999, 6 = USD $35,000–$49,999, 7 = USD $50,000–$74,999, and 8 = >USD $75,000); education (Coded: 1 = Less than high school, 2 = Some high school, 3 = Completed high school (or equivalent), 4 = Some college, 5 = Associate’s Degree, 6 = Bachelor’s Degree, 7 = Master’s Degree, 8 = Doctoral Degree, and 9 = More than Doctorate); and cigarettes smoked per day. Pain severity and perceived discrimination were then entered simultaneously at Step 2. The interaction between pain severity and perceived discrimination was entered at Step 3. The model fit for each of the steps was evaluated with the *F* statistic and change in *R*^2^. Squared semi-partial correlations (*sr*^2^) were used as indices of the effect size (interpreted as 0.01 = small, 0.09 = moderate, and 0.25 = large) [52]. Planned post hoc simple slope analyses were conducted with the PROCESS macro [53] to evaluate the relationship between pain severity with smoking abstinence expectancies at high and low levels of perceived discrimination (+/- 1 standard deviation from the mean). 

## 3. Results

The descriptive statistics and bivariate correlations are presented in Table 1. Pain severity was positively correlated with perceived discrimination, as well as negative mood, somatic symptoms, and harmful consequences abstinence expectancies (*r*’s range = 0.372–0.529). Pain severity was negatively correlated with cigarettes smoked per day (*r* = −0.182) and positive consequences abstinence expectancies (*r* = −0.492). Perceived discrimination was positively correlated with education (*r* = 0.205), as well as negative mood, somatic symptoms, and harmful consequences abstinence expectancies (*r*’s range = 0.446–0.565). Perceived discrimination was negatively correlated with age (*r* = −0.136), sex (i.e., lower levels of perceived discrimination were associated with being a female; *r* = −0.145), and positive consequences abstinence expectancies (*r* = −0.557).

### Hierarchical Regression Analyses 

**Figure 1:**
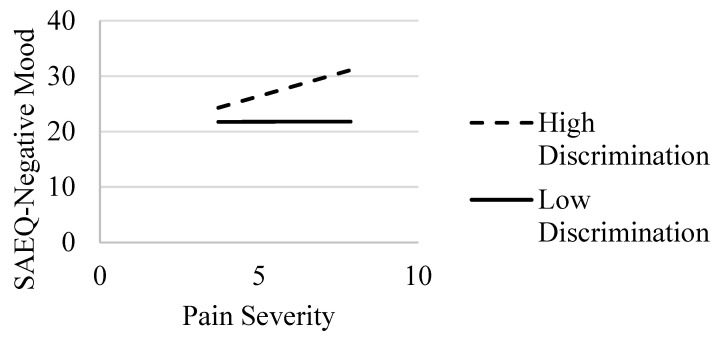
Interaction of pain severity and discrimination: association with SAEQ—negative mood subscale. Note. SAEQ = Smoking Abstinence Expectancies Questionnaire—negative mood subscale [22]. Effects of pain severity on SAEQ—negative mood at high (dashed line) and low (solid line) values of discrimination.

For the model with negative mood abstinence expectancies, Step 1 with covariates only was not statistically significant (*R*^2^ = 0.05, *F*(6, 219) = 1.79, *p* = 0.102). In Step 2, with the addition of pain severity and perceived discrimination added to the model, there was a statistically significant increase in variance accounted for (*ΔR*^2^ = 0.19, *F*(2, 217) = 27.58, *p* < 0.001), with the main effects for pain severity and perceived discrimination (*p* < 0.01) (see Table 2). In Step 3, the interaction term was added and accounted for a statistically significant increase in variance (*ΔR*^2^ = 0.04, *F*(1, 216) = 11.35, *p* < 0.001). The post hoc simple slope analysis revealed that pain severity was related to greater levels of negative mood abstinence expectancies for individuals with higher (*b* = 1.64, *SE* = 0.38, *p* < 0.001) but not lower (*b* = 0.02, *SE* = 0.41, *p* = 0.970) levels of perceived discrimination (see Figure 1). 

**Figure 2:**
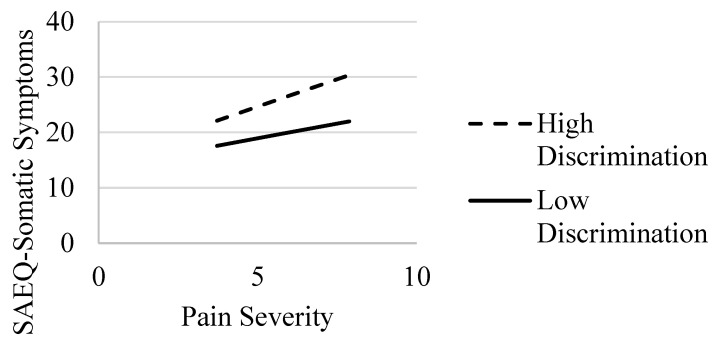
Interaction of pain severity and discrimination: Association with SAEQ—somatic symptoms subscale. Note. SAEQ = Smoking Abstinence Expectancies Questionnaire—somatic symptoms subscale [22]. Effects of pain severity on SAEQ—somatic symptoms at high (dashed line) and low (solid line) values of discrimination.

In the model with somatic symptoms abstinence expectancies, Step 1 with covariates only was statistically significant (*R*^2^ = 0.13, *F*(6, 219) = 5.28, *p* < 0.001). Examining the individual clinical correlates revealed that age and education were statistically significant predictors of somatic symptoms abstinence expectancies. In Step 2, with pain severity and perceived discrimination added to the model, there was a statistically significant increase in explained variance (*ΔR*^2^ = 0.32, *F*(2, 217) = 61.03, *p* < 0.001); the main effects were evident for pain severity and perceived discrimination (*p* < 0.001) (see Table 3). With the addition of the interaction term in Step 3, there was a statistically significant increase in *R*^2^ (*ΔR*^2^ = 0.01, *F*(1, 216) = 4.58, *p* = 0.033). The simple slope analysis revealed that pain severity was related to greater levels of somatic symptoms abstinence expectancies, with associations stronger for those with higher (*b* = 1.98, *SE* = 0.34, *p* < 0.001) relative to lower (*b* = 1.06, *SE* = 0.37, *p* = 0.004) levels of perceived discrimination (see Figure 2). 

**Figure 3:**
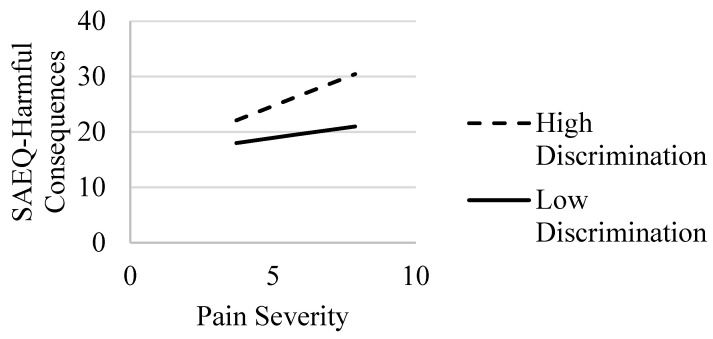
Interaction of pain severity and discrimination: association with the SAEQ—harmful consequences subscale. Note. SAEQ = Smoking Abstinence Expectancies Questionnaire—harmful consequences subscale [22]. Effects of pain severity on the SAEQ—harmful consequences at high (dashed line) and low (solid line) values of discrimination.

In the model with harmful consequences abstinence expectancies, Step 1 with covariates only was statistically significant (*R*^2^ = 0.07, *F*(6, 219) = 2.74, *p* = 0.014). Examining the individual clinical correlates revealed that age and education were statistically significant predictors of harmful consequences abstinence expectancies. In Step 2 of the model, with the addition of pain severity and perceived discrimination, there was a statistically significant increase in *R*^2^ (*ΔR*^2^ = 0.30, *F*(2, 217) = 51.66, *p* < 0.001); the main effects were evident for pain severity and perceived discrimination (*p* < 0.001) (see Table 4). With the addition of the interaction term in Step 3, there was a statistically significant increase in variance explained (*ΔR*^2^ = 0.02, *F*(1, 216) = 7.91, *p* = 0.005). The post hoc simple slope analysis demonstrated that pain severity was related to greater levels of harmful consequences abstinence expectancies for individuals with higher (*b* = 2.01, *SE* = 0.36, *p* < 0.001) but not lower (*b* = 0.72, *SE* = 0.39, *p* = 0.065) levels of perceived discrimination (see Figure 3). 

**Figure 4:**
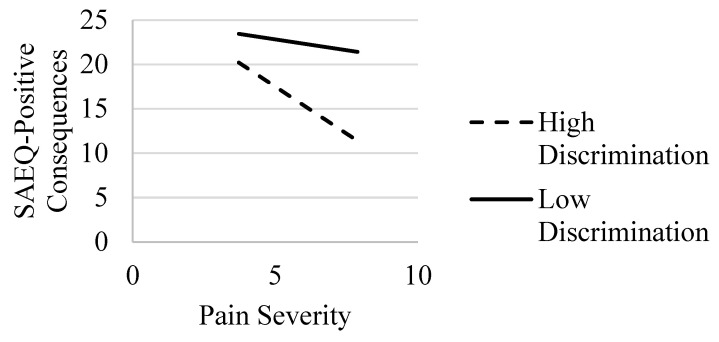
Interaction of Pain Severity and Discrimination: association with the SAEQ—positive consequences subscale. Note. SAEQ = Smoking Abstinence Expectancies Questionnaire—positive consequences subscale [22]. Effects of pain severity on the SAEQ—positive consequences at high (dashed line) and low (solid line) values of discrimination.

For the model with positive consequences abstinence expectancies, Step 1 of the model with covariates was statistically significant (*R*^2^ = 0.13, *F*(6, 219) = 5.65, *p* < 0.001); age and education were statistically significant predictors. In Step 2 with the addition of pain severity and perceived discrimination added to the model, there was a statistically significant increase in the variance explained (*ΔR*^2^ = 0.28, *F*(2, 217) = 52.80, *p* < 0.001) with the main effects for pain severity and discrimination (*p* < 0.001) (see Table 5). In Step 3, the interaction term was added and accounted for a statistically significant increase in the variance that was explained (*ΔR*^2^ = 0.04, *F*(1, 216) = 13.93, *p* < 0.001). The post hoc simple slope analysis revealed that pain severity was related to lower levels of positive consequences abstinence expectancies for individuals with higher (*b* = −2.12, *SE* = 0.35, *p* < 0.001) but not lower (*b* = −0.49, *SE* = 0.37, *p* = 0.189) levels of perceived discrimination (see Figure 4). 

## 4. Discussion

The current study sought to examine the interactive association between pain severity and perceived discrimination in relation to smoking abstinence expectancies among Latinx individuals who currently smoke cigarettes. The results were consistent with predictions in that associations between pain severity and each smoking abstinence expectancy (e.g., negative mood, somatic symptoms, harmful consequences, and positive consequences) were moderated by perceived discrimination. Specifically, the associations of pain severity on smoking abstinence expectancies related to negative mood, harmful consequences, and positive consequences were evident for individuals with higher, but not lower, perceived discrimination. Additionally, associations between pain severity and somatic symptom smoking abstinence expectancies were stronger for individuals with higher, relative to lower, perceived discrimination. Although evident even after accounting for a range of relevant covariates, including age, sex, nativity, income, education, and number of cigarettes smoked per day, the observed effect sizes were generally small across the models (*sr^2^* range = 0.012–0.038). These findings add to an extant literature highlighting the negative effects of perceived discrimination on pain-related outcomes among Latinx individuals [29,34,35] and extend these models to smoking-related processes.

The current findings are consistent with the fear–avoidance model of pain [54,55] in that pain severity may increase fear and avoidance behaviors [56], including beliefs about the effects of abstaining from cigarette smoking (e.g., “I would feel tense”). These relations appear to be evident (or stronger in some cases, i.e., somatic symptom smoking abstinence expectancies) at higher levels of perceived discrimination, consistent with minority stress models implicating monitory stress as a salient contributor to poorer behavioral health outcomes [57]. The interactive association between perceived discrimination and pain severity in terms of smoking abstinence expectancies may be due to a variety of factors. For example, perceived discrimination can increase psychological distress [35] and may amplify pain experiences [29,34], resulting in an allostatic overload [58]. Cigarette smoking may also be employed in an effort to escape and/or avoid such distressing experiences [8,59,60], thus impeding or delaying pain-related treatment seeking [11,12,36]. Over time, repeated patterns of a learned behavior (i.e., utilizing smoking to cope with pain) may inform the development of beliefs regarding the anticipated effects of smoking abstinence. 

It is important to consider the current results in terms of potential clinical implications and societal impact. In the context of cessation interventions for Latinx individuals who smoke cigarettes, culturally sensitive approaches may benefit from addressing not only pain severity, which tends to be amplified among Latinx individuals [10,11], but also the impact of perceived discrimination on pain experience and the maintenance of smoking [8,59,60]. Moreover, Latinx individuals report perceived discrimination from healthcare providers as a deterrent to seeking treatment for pain [11,12,36]. Thus, clinical treatment strategies, including proactive care interventions designed to overcome barriers to smoking cessation treatments [61] and mobile health interventions [62], may be warranted to address the needs of Latinx individuals with co-occurring pain who continue to smoke cigarettes. 

Interestingly, findings from the current study indicate pain severity was negatively correlated with the number of cigarettes smoked per day. This may be due to the use of cigarette smoking as a means to cope with pain experiences [13]. As such, a reduction in cigarette smoking, in the absence of alternative coping methods, may increase pain severity. However, it is important to note that these findings are correlational and weak in magnitude. Nonetheless, the current findings may have clinical implications. For example, Latinx individuals who smoke cigarettes may benefit from psychoeducation on alternative coping mechanisms for pain management (e.g., exercise) [63] when reducing cigarette smoking. However, longitudinal works examining the association between pain severity and cigarettes smoked per day are needed to determine if the reduction in cigarettes smoked per day precedes greater pain severity.

The current study has several limitations that are also worth noting. First, these data are cross-sectional, and all constructs were assessed at one time point, thus prohibiting inferences regarding causation and directionality. Second, the majority of participants (86.3%) were born in the US, which compares to national estimates indicating that 55.2% of Latinx adults are born in the US [64]. Future studies may benefit from collecting data from a more diverse group of Latinx individuals in terms of nativity to enhance the generalizability to the US population. Third, participants were not selected based on pain status. Thus, additional research is needed to explore the current relations among a sample of Latinx individuals with chronic pain who smoke cigarettes. Finally, all measures were collected via self-reporting, and future works may benefit from employing additional methodologies such as laboratory paradigms (e.g., cold pressor and cyberball task) [65,66] to further explore the proposed relations. 

## 5. Conclusions

Overall, the current study provides initial support for the interactive association between perceived discrimination and pain severity in terms of smoking abstinence expectancies related to negative mood, somatic symptoms, harmful consequences, and positive consequences. These findings suggest that greater pain severity, in the context of higher perceived discrimination, is associated with smoking abstinence expectancies that portend less successful cessation-related outcomes (e.g., greater nicotine dependence, withdrawal severity, etc.) [22]. Future works are needed to explore longitudinal associations between these constructs and implications for smoking cessation outcomes. Moreover, additional research is needed to isolate the impact of social determinants of health on smoking cessation-relevant behavioral health outcomes among Latinx individuals.

## Figures and Tables

**Table 1:** Correlations among the study variables.

Variable	*M/%* (*SD/N*)	1	2	3	4	5	6	7	8	9	10	11	12
1. Age	34.95 (8.62)	-											
2. Sex	38.5% (87)	0.003	-										
3. Nativity	13.7% (31)	0.112	0.081	-									
4. Income	18.1% (41)	0.150 *	−0.254 ***	−0.021	-								
5. Education	81.9% (185)	0.111	−0.271 ***	0.004	0.533 ***	-							
6. Cigarettes per Day	12.15 (10.37)	0.052	−0.102	−0.085	−0.059	−0.019	-						
7. Pain Severity	5.80 (2.08)	0.006	−0.074	0.069	0.039	0.124	−0.182 **	-					
8. Discrimination	3.11 (1.01)	−0.136 *	−0.145 *	−0.030	0.099	0.205 **	−0.117	0.517 ***	-				
9. SAEQ—Negative Mood	25.61 (9.45)	−0.053	−0.063	−0.093	0.067	0.150 *	−0.103	0.372 ***	0.446***	-			
10. SAEQ—Somatic Symptoms	23.51 (9.72)	−0.134 *	−0.164 *	0.007	0.194 **	0.280 ***	−0.102	0.529 ***	0.565***	0.739 ***	-		
11.SAEQ—Harmful Consequences	23.59 (9.80)	−0.118	−0.085	−0.021	0.120	0.208 **	−0.082	0.489 ***	0.540***	0.821 ***	0.910 ***	-	
12. SAEQ—Positive Consequences	18.25 (9.86)	0.149 *	0.161 *	0.021	−0.200 **	−0.280 ***	0.116	−0.492 ***	−0.557***	−0.618 ***	−0.815 ***	−0.778 ***	-

Note. * *p* < 0.05, ** *p* < 0.01, and *** *p*< 0.001. Sex % listed as females (Coded: 0 = Male, 1 = Female); Nativity % listed as Born Outside of the US (Coded: 0 = US Born, 1 = Born Outside of US); Income % listed as <USD $25,000 annual income (Coded 1 = USD $0–$4999, 2 = USD $5000–$9999, 3 = USD $10,000–$14,999, 4 = USD $15,000–$24,999, 5 = USD $25,000–$34,999, 6 = USD $35,000–$49,999, 7 = USD $50,000–$74,999, and 8 = >USD $75,000); Education % listed as at least some college (Coded: 1 = Less than high school, 2 = Some high school, 3 = Completed high school (or equivalent), 4 = Some college, 5 = Associate’s Degree, 6 = Bachelor’s Degree, 7 = Master’s Degree, 8 = Doctoral Degree, and 9 = More than Doctorate); and SAEQ = Smoking Abstinence Expectancies Questionnaire and the Negative Mood, Somatic Symptoms, Harmful Consequences, and Positive Consequences subscales [22].

**Table 2:** Hierarchical regression results for the Smoking Abstinences Expectancies Questionnaire—negative mood subscale.

Model		*b*	*SE*	*β*	*t*	*p*	*CI (l)*	*CI (u)*	*sr^2^*
1	Age	−0.06	0.07	−0.05	−0.75	0.456	−0.20	0.09	0.002
	Sex	−0.58	1.36	−0.03	−0.42	0.672	−3.25	2.10	0.001
	Nativity	−2.61	1.83	−0.10	−1.42	0.156	−6.22	1.01	0.009
	Income	−0.13	0.39	−0.03	−0.33	0.742	−0.89	0.63	<0.001
	Education	0.94	0.47	0.16	2.01	0.045	0.02	1.86	0.018
	Cigarettes Per Day	−0.10	0.06	−0.11	−1.64	0.103	−0.22	0.02	0.012
2	Age	0.00	0.07	0.00	−0.05	0.959	−0.14	0.13	<0.001
	Sex	0.39	1.22	0.02	0.32	0.750	−2.02	2.80	<0.001
	Nativity	−2.77	1.65	−0.10	−1.68	0.095	−6.02	0.48	0.010
	Income	−0.04	0.35	−0.01	−0.10	0.918	−0.72	0.65	<0.001
	Education	0.40	0.42	0.07	0.94	0.350	−0.44	1.24	0.003
	Cigarettes Per Day	−0.03	0.06	−0.03	−0.56	0.577	−0.14	0.08	0.001
	Pain Severity	0.90	0.32	0.20	2.80	0.006	0.27	1.53	0.027
	Discrimination	3.04	0.67	0.33	4.54	<0.001	1.72	4.37	0.072
3	Age	0.02	0.07	0.02	0.31	0.760	−0.11	0.15	<0.001
	Sex	0.84	1.20	0.04	0.70	0.488	−1.53	3.20	0.002
	Nativity	−2.45	1.61	−0.09	−1.52	0.131	−5.63	0.74	0.008
	Income	−0.12	0.34	−0.02	−0.36	0.722	−0.79	0.55	<0.001
	Education	0.22	0.42	0.04	0.53	0.595	−0.60	1.05	0.001
	Cigarettes Per Day	−0.06	0.06	−0.06	−1.04	0.301	−0.17	0.05	0.004
	Pain Severity	−1.67	0.82	−0.37	−2.03	0.044	−3.29	−0.05	0.014
	Discrimination	−1.72	1.56	−0.18	−1.10	0.271	−4.79	1.35	0.004
	Pain Severity × Discrimination	0.80	0.24	0.96	3.37	0.001	0.33	1.27	0.038

Note. Sex (Coded: 0 = Male, 1 = Female); Nativity (Coded: 0 = US Born, 1 = Born Outside of US); Income (Coded 1 = USD $0–$4999, 2 = USD $5000–$9999, 3 = USD $10,000–$14,999, 4 = USD $15,000–$24,999, 5 = USD $25,000–$34,999, 6 = USD $35,000–$49,999, 7 = USD $50,000–$74,999, and 8 = >USD $75,000); and Education (Coded: 1 = Less than high school, 2 = Some high school, 3 = Completed high school (or equivalent), 4 = Some college, 5 = Associate’s Degree, 6 = Bachelor’s Degree, 7 = Master’s Degree, 8 = Doctoral Degree, and 9 = More than Doctorate).

**Table 3:** Hierarchical regression results for the Smoking Abstinences Expectancies Questionnaire—somatic symptoms subscale.

Model		*b*	*SE*	*β*	*t*	*p*	*CI (l)*	*CI (u)*	*sr^2^*
1	Age	−0.19	0.07	−0.17	−2.59	0.010	−0.33	−0.05	0.027
	Sex	−1.90	1.33	−0.10	−1.42	0.157	−4.53	0.73	0.008
	Nativity	0.73	1.81	0.03	0.41	0.685	−2.83	4.29	0.001
	Income	0.32	0.38	0.06	0.85	0.398	−0.43	1.07	0.003
	Education	1.43	0.46	0.24	3.12	0.002	0.53	2.34	0.039
	Cigarettes Per Day	−0.09	0.06	−0.09	−1.44	00.150	−0.21	0.03	0.008
2	Age	−0.13	0.06	−0.12	−2.26	0.025	−0.25	−0.02	0.013
	Sex	−0.67	1.08	−0.03	−0.62	0.537	−2.79	1.46	0.001
	Nativity	0.36	1.46	0.01	0.24	0.807	−2.51	3.22	<0.001
	Income	0.46	0.31	0.09	1.50	0.135	−0.14	1.06	0.006
	Education	0.75	0.37	0.12	2.01	0.046	0.01	1.49	0.010
	Cigarettes Per Day	0.01	0.05	0.01	0.19	0.849	−0.09	0.11	<0.001
	Pain Severity	1.56	0.28	0.33	5.52	<0.001	1.00	2.12	0.078
	Discrimination	3.25	0.59	0.34	5.51	<0.001	2.09	4.42	0.078
3	Age	−0.12	0.06	−0.11	−2.03	0.043	−0.24	0.00	0.010
	Sex	−0.41	1.08	−0.02	−0.38	0.702	−2.53	1.71	<0.001
	Nativity	0.54	1.45	0.02	0.37	0.710	−2.31	3.39	<0.001
	Income	0.41	0.30	0.08	1.35	0.179	−0.19	1.01	0.005
	Education	0.65	0.37	0.11	1.74	0.083	−0.09	1.39	0.008
	Cigarettes Per Day	−0.01	0.05	−0.01	−0.11	0.914	−0.10	0.09	<0.001
	Pain Severity	0.10	0.74	0.02	0.14	0.892	−1.35	1.55	<0.001
	Discrimination	0.54	1.40	0.06	0.39	0.698	−2.21	3.29	<0.001
	Pain Severity × Discrimination	0.46	0.21	0.53	2.14	0.033	0.04	0.88	0.012

Note. Sex (Coded: 0 = Male, 1 = Female); Nativity (Coded: 0 = US Born, 1 = Born Outside of US); Income (Coded 1 = USD $0–$4999, 2 = USD $5000–$9999, 3 = USD $10,000–$14,999, 4 = USD $15,000–$24,999, 5 = USD $25,000–$34,999, 6 = USD $35,000–$49,999, 7 = USD $50,000–$74,999, and 8 = >USD $75,000); and Education (Coded: 1 = Less than high school, 2 = Some high school, 3 = Completed high school (or equivalent), 4 = Some college, 5 = Associate’s Degree, 6 = Bachelor’s Degree, 7 = Master’s Degree, 8 = Doctoral Degree, and 9 = More than Doctorate).

**Table 4:** Hierarchical regression results for the Smoking Abstinences Expectancies Questionnaire—harmful consequences subscale.

Model		*b*	*SE*	*β*	*t*	*p*	*CI (l)*	*CI (u)*	*sr^2^*
1	Age	−0.16	0.08	−0.14	−2.08	0.038	−0.31	−0.01	0.018
	Sex	−0.63	1.39	−0.03	−0.45	0.652	−3.36	2.11	0.001
	Nativity	−0.28	1.88	−0.01	−0.15	0.881	−3.98	3.42	<0.001
	Income	0.10	0.40	0.02	0.25	0.799	−0.68	0.88	<0.001
	Education	1.24	0.48	0.20	2.59	0.010	0.30	2.18	0.029
	Cigarettes Per Day	−0.07	0.06	−0.07	−1.12	0.265	−0.19	0.05	0.005
2	Age	−0.10	0.06	−0.09	−1.56	0.119	−0.22	0.03	0.007
	Sex	0.60	1.15	0.03	0.52	0.606	−1.68	2.87	0.001
	Nativity	−0.60	1.56	−0.02	−0.39	0.699	−3.67	2.47	<0.001
	Income	0.23	0.33	0.05	0.70	0.482	−0.42	0.88	0.001
	Education	0.56	0.40	0.09	1.39	0.166	−0.23	1.35	0.006
	Cigarettes Per Day	0.02	0.05	0.02	0.44	0.660	−0.08	0.13	0.001
	Pain Severity	1.42	0.30	0.30	4.69	<0.001	0.82	2.02	0.064
	Discrimination	3.44	0.63	0.36	5.44	<0.001	2.20	4.69	0.086
3	Age	−0.08	0.06	−0.07	−1.28	0.201	−0.20	0.04	0.005
	Sex	0.95	1.14	0.05	0.83	0.406	−1.30	3.21	0.002
	Nativity	−0.35	1.54	−0.01	−0.23	0.821	−3.38	2.68	<0.001
	Income	0.16	0.32	0.03	0.50	0.615	−0.47	0.80	0.001
	Education	0.42	0.40	0.07	1.05	0.294	−0.37	1.20	0.003
	Cigarettes Per Day	0.00	0.05	0.00	0.05	0.960	−0.10	0.11	<0.001
	Pain Severity	−0.62	0.78	−0.13	−0.79	0.431	−2.16	0.93	0.002
	Discrimination	−0.34	1.48	−0.04	−0.23	0.818	−3.27	2.58	<0.001
	Pain Severity × Discrimination	0.64	0.23	0.74	2.81	0.005	0.19	1.08	0.022

Note. Sex (Coded: 0 = Male, 1 = Female); Nativity (Coded: 0 = US Born, 1 = Born Outside of US); Income (Coded 1 = USD $0–$4999, 2 = USD $5000–$9999, 3 = USD $10,000–$14,999, 4 = USD $15,000–$24,999, 5 = USD $25,000–$34,999, 6 = USD $35,000–$49,999, 7 = USD $50,000–$74,999, and 8 = >USD $75,000); and Education (Coded: 1 = Less than high school, 2 = Some high school, 3 = Completed high school (or equivalent), 4 = Some college, 5 = Associate’s Degree, 6 = Bachelor’s Degree, 7 = Master’s Degree, 8 = Doctoral Degree, and 9 = More than Doctorate).

**Table 5:** Hierarchical regression results for the Smoking Abstinences Expectancies Questionnaire—positive consequences subscale.

Model		*b*	*SE*	*β*	*t*	*p*	*CI (l)*	*CI (u)*	*sr^2^*
1	Age	0.21	0.07	0.18	2.80	0.006	0.06	0.35	0.031
	Sex	1.80	1.35	0.09	1.33	0.183	−0.86	4.46	0.007
	Nativity	0.06	1.82	0.00	0.03	0.972	−3.53	3.66	<0.001
	Income	−0.37	0.38	−0.07	−0.97	0.332	−1.13	0.38	0.004
	Education	−1.44	0.46	−0.23	−3.11	0.002	−2.35	−0.53	0.038
	Cigarettes Per Day	0.10	0.06	0.11	1.67	0.096	−0.02	0.22	0.011
2	Age	0.15	0.06	0.13	2.41	0.017	0.03	0.27	0.016
	Sex	0.60	1.12	0.03	0.54	0.592	−1.60	2.80	0.001
	Nativity	0.37	1.51	0.01	0.25	0.806	−2.60	3.34	<0.001
	Income	−0.50	0.32	−0.10	−1.58	0.116	−1.13	0.12	0.007
	Education	−0.77	0.39	−0.13	−2.00	0.047	−1.54	−0.01	0.011
	Cigarettes Per Day	0.01	0.05	0.01	0.21	0.834	−0.09	0.11	<0.001
	Pain Severity	−1.37	0.29	−0.29	−4.70	<0.001	−1.95	−0.80	0.059
	Discrimination	−3.40	0.61	−0.35	−5.55	<0.001	−4.60	−2.19	0.083
3	Age	0.12	0.06	0.11	2.07	0.039	0.01	0.24	0.011
	Sex	0.15	1.09	0.01	0.14	0.890	−2.00	2.30	<0.001
	Nativity	0.05	1.47	0.00	0.03	0.975	−2.84	2.94	<0.001
	Income	−0.41	0.31	−0.08	−1.34	0.181	−1.02	0.19	0.005
	Education	−0.60	0.38	−0.10	−1.57	0.117	−1.35	0.15	0.006
	Cigarettes Per Day	0.04	0.05	0.04	0.74	0.463	−0.06	0.14	0.001
	Pain Severity	1.21	0.75	0.25	1.61	0.108	−0.27	2.68	0.007
	Discrimination	1.40	1.42	0.14	0.99	0.324	−1.39	4.19	0.002
	Pain Severity × Discrimination	−0.81	0.22	−0.93	−3.73	<0.001	−1.23	−0.38	0.035

Note. Sex (Coded: 0 = Male, 1 = Female); Nativity (Coded: 0 = US Born, 1 = Born Outside of US); Income (Coded 1 = USD $0–$4999, 2 = USD $5000–$9999, 3 = USD $10,000–$14,999, 4 = USD $15,000–$24,999, 5 = USD $25,000–$34,999, 6 = USD $35,000–$49,999, 7 = USD $50,000–$74,999, and 8 = >USD $75,000); and Education (Coded: 1 = Less than high school, 2 = Some high school, 3 = Completed high school (or equivalent), 4 = Some college, 5 = Associate’s Degree, 6 = Bachelor’s Degree, 7 = Master’s Degree, 8 = Doctoral Degree, and 9 = More than Doctorate).

## Data Availability

The data presented in this study are available on request from the corresponding author. The data are not publicly available due to restrictions from the institution.
